# Phenotypic correlates of the working dog microbiome

**DOI:** 10.1038/s41522-022-00329-5

**Published:** 2022-08-22

**Authors:** Hillary A. Craddock, Anastasia Godneva, Daphna Rothschild, Yair Motro, Dan Grinstein, Yuval Lotem-Michaeli, Tamar Narkiss, Eran Segal, Jacob Moran-Gilad

**Affiliations:** 1 MAGICAL Group, Department of Health Policy and Management, Ben-Gurion University of the Negev, Beer-Sheva, Israel; 2 Department of Computer Science and Applied Math, Weizmann Institute of Science, Rehovot, Israel; 3 Israel Defense Forces Medical Corps, Tel Hashomer, Ramat Gan, Israel

**Keywords:** Metagenomics, Microbiome, Environmental microbiology

## Abstract

Dogs have a key role in law enforcement and military work, and research with the goal of improving working dog performance is ongoing. While there have been intriguing studies from lab animal models showing a potential connection between the gut microbiome and behavior or mental health there is a dearth of studies investigating the microbiome-behavior relationship in working dogs. The overall objective of this study was to characterize the microbiota of working dogs and to determine if the composition of the microbiota is associated with behavioral and performance outcomes. Freshly passed stools from each working canine (Total *n* = 134) were collected and subject to shotgun metagenomic sequencing using Illumina technology. Behavior, performance, and demographic metadata were collected. Descriptive statistics and prediction models of behavioral/phenotypic outcomes using gradient boosting classification based on Xgboost were used to study associations between the microbiome and outcomes. Regarding machine learning methodology, only microbiome features were used for training and predictors were estimated in cross-validation. Microbiome markers were statistically associated with motivation, aggression, cowardice/hesitation, sociability, obedience to one trainer vs many, and body condition score (BCS). When prediction models were developed based on machine learning, moderate predictive power was observed for motivation, sociability, and gastrointestinal issues. Findings from this study suggest potential gut microbiome markers of performance and could potentially advance care for working canines.

## Introduction

Dogs have a key role in law enforcement and military work, and research with the goal of improving working dog performance is ongoing. While there have been intriguing studies from lab animal models showing a potential connection between the gut microbiome and behavior or mental health, implicating the so-called “gut-brain axis”^1,2^, there is a dearth of studies investigating the microbiome-behavior relationship in working dogs. Some recent small-scale studies in non-working dogs have found that undesirable canine behaviors (i.e., aggression, anxiety) are associated with certain characteristics of the canine gut microbiome^3,4^. However, it is critical to note when thinking about the microbiome’s impact on behavior that the behavioral traits of working dogs and companion dogs have critical differences^5^. Thus, focusing on working dogs is important for understanding potential microbiome-behavior connections as it pertains to job performance and potentially modulating the microbiome to influence performance outcomes.

Significant efforts have been made to characterize the canine microbiome of companion animals and lab animals in both health and disease^6–23^. Dominant phyla include *Firmicutes, Proteobacteria, Fusobacteria, Bacteroides*, and *Actinobacteria*, however, abundancies and the most prevalent of these phyla differ among studies^8,16,19,24,25^. Canine microbiome dysbiosis has been observed in conditions including inflammatory bowel disease, acute diarrhea, skin and ear infection, and obesity^6,7,10,13,15,17,18,20–23,26,27^.

Despite these efforts to characterize the canine microbiome, there are strikingly few studies investigating the impact of the microbiome on canine performance in general and working dog performance in particular. One review article postulated that there could be a connection between the gut microbiome and olfaction performance for scent-detection dogs^28^, and one study observed small differences in nasal and oral microbiota among different types of scent-detection dogs^29^. Two pilot studies investigating the impact of the stress of helicopter and flight travel on the performance and microbiome of United States Federal Emergency Management Agency search and rescue (SAR) dogs found a difference in microbiome after plane travel but not helicopter travel^30,31^.

Furthermore, current research is limited regarding the canine microbiota and has for the most part utilized 16S rRNA amplicon sequencing, and studies regarding the microbiome of working canines as well as microbiome-behavior connections have had small sample sizes^28–31^. Furthermore, even in the heavily-studied human gut there are still unknowns regarding the composition and functionality of the microbiome^32,33^, and this knowledge gap is wider in the relatively understudied microbiome of large mammals such as canines.

The overall objective of this study was to characterize the microbiota of working dogs across a sample of 134 working dogs representing diversity with respect to age, sex, breed, job, and behavioral traits, and to determine if the composition of the microbiota are associated with behavioral and performance outcomes. By harnessing whole genome metagenomic methods and predictive modeling, we sought to comprehensively characterize the working canine microbiota and its potential impact on behavior and performance.

## Results

### Quantitative phenotypes description

The age range of sampled dogs was 0.5–12 years (mean 3.8 years, median 3.4 years), and slightly over half were females (53.4%). BCS ranged from 4 to 6.5 on a scale of 1–9 (mean 4.95, median 5). The most common breed was Malinois at 43.5%, followed by non-Malinois Belgian and Dutch shepherds at 18.5%. In respect to dog’s job assignments 29.5% were tracking dogs and 18.8% were breeding dogs, followed by scent detection, bite work, and SAR dogs (14.7, 13.3, and 12.3% respectively). Job differed by breed (*p* = 0.0005) and sex (*p* = 0.003). Some demographic variables varied between cohorts, notably breed (*p* < 0.0001) and job (*p* < 0.0001). For example, the most common breed in both cohorts was the Belgian Malinois (Cohort 1–50%, Cohort 2–29.7%); however the second most common breed in the first cohort was non-Malinois Belgian and Dutch Shepherds (31.9%), and the second most-common breed in the second cohort was the German Shepherd (26.6%). The most common jobs in the first cohort were tracking (48.6%) followed by scent detection (13.9%); the most common jobs in the second cohort were breeding (32.8%) followed by SAR and bite work (both 20.3% of the cohort). There were 9 dogs who received a special diet (hypoallergenic food), 4 were treated with proton pump inhibitors, and 18 from cohort 1 had received antibiotics between 1 month and 1 year prior to sample collection. Further demographic information is depicted in Table 1. Overall, *Bacteroides* and *Firmicutes* were the most commonly observed phyla; in Cohort 1 *Bacteroides* were more abundant than *Firmicutes* and in Cohort 2 *Firmicutes* were observed to be more abundant than *Bacteroides* (Fig. S1 and Table S1). Principle coordinates analysis (PCoA) plots detailing beta diversity by year, breed, and year, and job and year are presented in Fig. S2.

**Table 1:** Demographics of sampled working canines (Total *n* = 134).

**Demographic characteristics**		
Sex	Female %	53.4
	Male %	46.6
Puppy/adult	Puppy %	11.8
	Adult %	88.2
Sterilized	No %	88.7
	Yes %	11.3
Chronic GI issues	Yes %	10.3
	No %	89.7
Breed	Malinois %	43.5
	Belgian/Dutch Shepherd %	18.5
	Corgi %	9.7
	GSD %	12.9
	Labrador %	6.4
	Other %	8.9
Job	Bite work %	13.1
	Breeding %	18.8
	Search and Rescue %	12.3
	Scent detection %	14.7
	Tracking %	29.5
	Failed %	11.5
Body condition score (scale)	Median 5 Range (4–6.5)	StDev 0.6
Age (years)	Mean 3.8 Range (0.5–12)	StDev 2.4
**Behavioral and performance characteristics**		
	Median (range)	StDev
Obedience	4 (2–5)	0.9
Obedience (specific)	5 (1–5)	1
Motivation	4 (2–5)	0.8
Aggression	1 (1–5)	1.1
Cowardice	2 (1–4)	0.8
Sociability	5 (2–5)	0.8
Stress level	2 (1–4)	0.9
Job performance	4 (1–5)	0.8

**Fig. 1:**
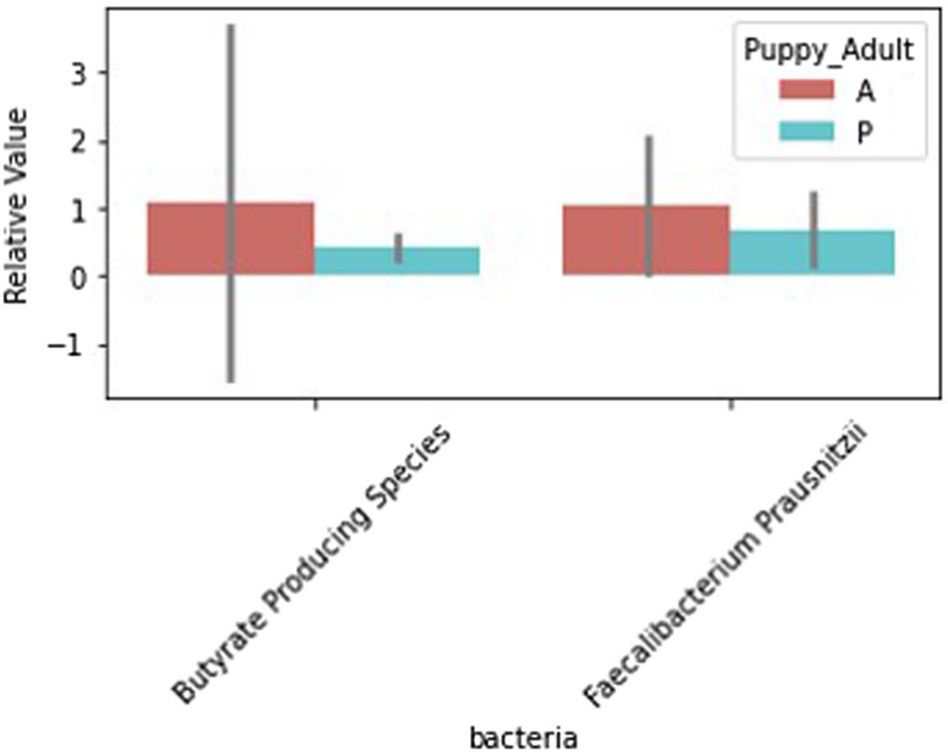
Relative abundance of microbiome features that differed by the age group (adult (A) vs puppy (P)) of working canines and are statistically significant (only associations significant via Mann–Whitney test, *p* < 0.05, are included). Error bars represent confidence intervals.

**Fig. 2:**
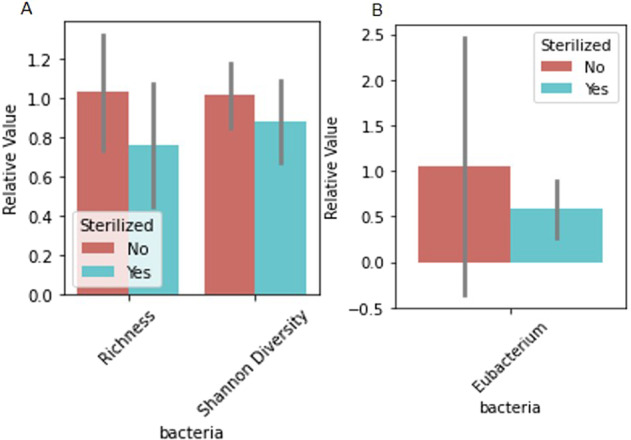
Relative value of microbiome features that differed by the sterilization status (Dog was spayed/neutered or not) of the dog and are statistically significant (only associations significant via by Mann–Whitney test, *p* < 0.05, are included). **A** Reflects differential value of richness and diversity, and **B** reflects relative abundance. Error bars represent confidence intervals.

**Fig. 3:**
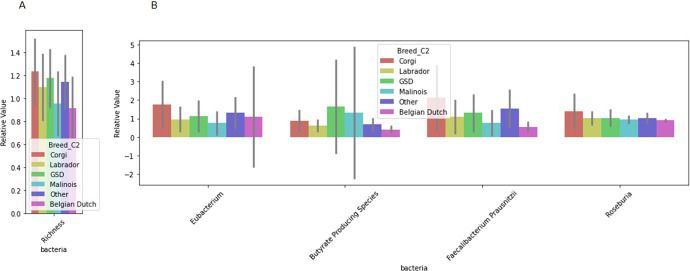
Relative value of microbiome features that differed by canine breed group and are statistically significant (only associations significant via by Kruskal test, *p* < 0.05, are included). **A** Reflects differential value of richness and diversity, and **B** reflects relative abundance. Error bars represent confidence intervals. *GSD German Shepherd Dog.

**Fig. 4:**
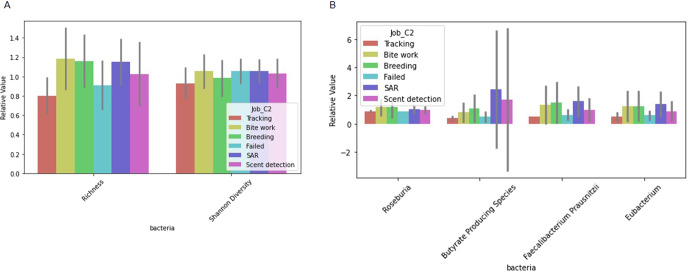
Relative value of microbiome features that differed by canine job group and are statistically significant (Only associations significant via by Kruskal test, *p* < 0.05, are included). **A** Reflects differential value of richness and diversity, and **B** reflects relative abundance. Error bars represent confidence intervals. *SAR Search and Rescue. **Failed = Dogs that failed out of job training or failed to be assigned to job training.

**Fig. 5:**
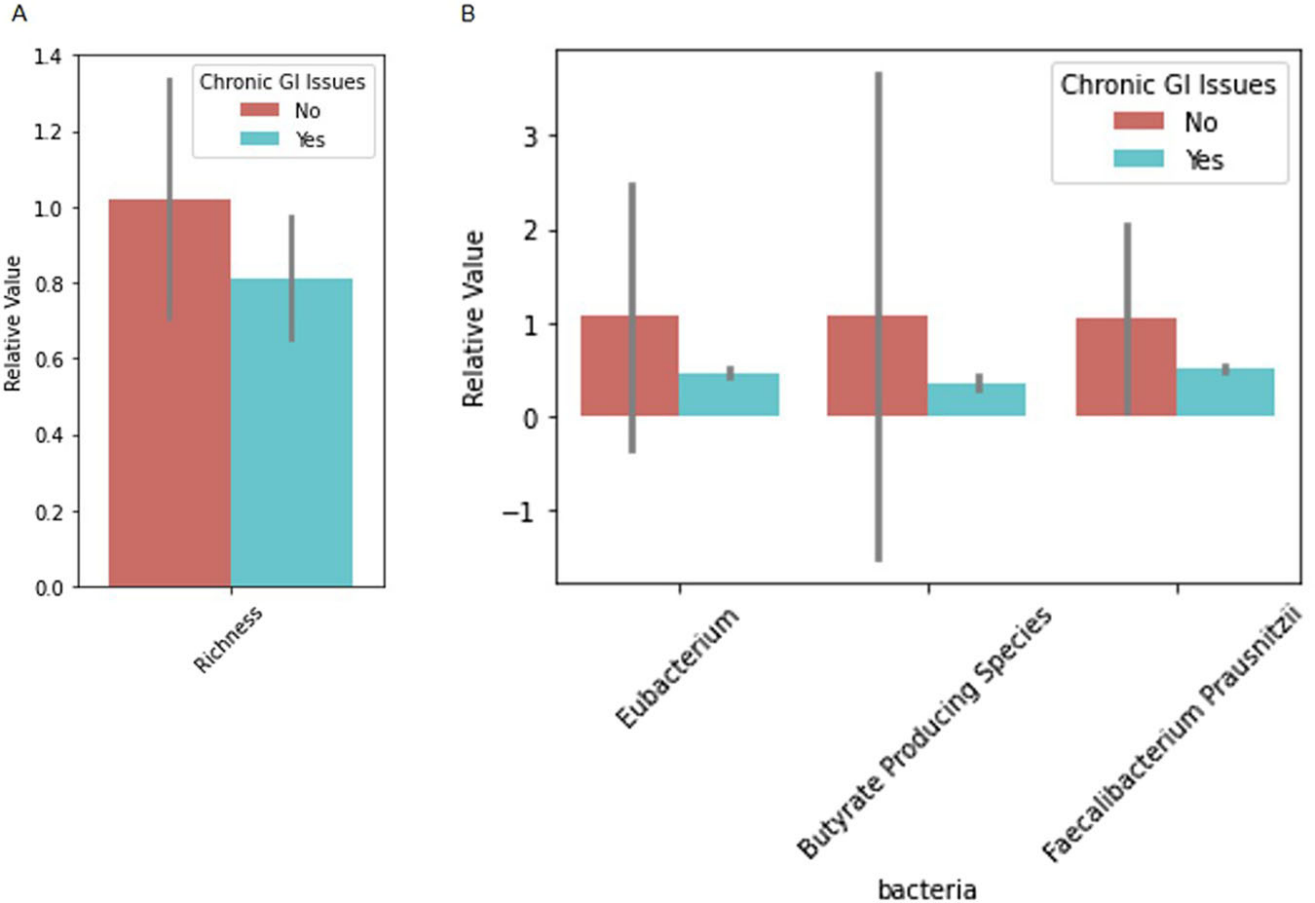
Relative value of microbiome features that differed by if the dog had recurrent gastrointestinal (GI) illness or not (only associations significant via Mann–Whitney test, *p* < 0.05 are included). **A** Reflects differential value of richness, and **B** reflects relative abundance. Error bars represent confidence intervals.

Differences in microbiome markers among demographic groups, including diet, age, sterilization status, breed, job, and if the dog had recurrent gastrointestinal (GI) issues, are presented in Figs. 1–5, S3–S4. For example, number of species as well as diversity were higher in dogs that had not been sterilized than dogs that had been sterilized (Fig. 2) and differed among different job groups (Fig. 4). Dogs with recurrent GI problems had a lower richness and lower abundance of Butyrate producing species than dogs without recurrent GI problems (Fig. 5). No significant relationships were observed between microbiome markers and sex.

**Fig. 6:**
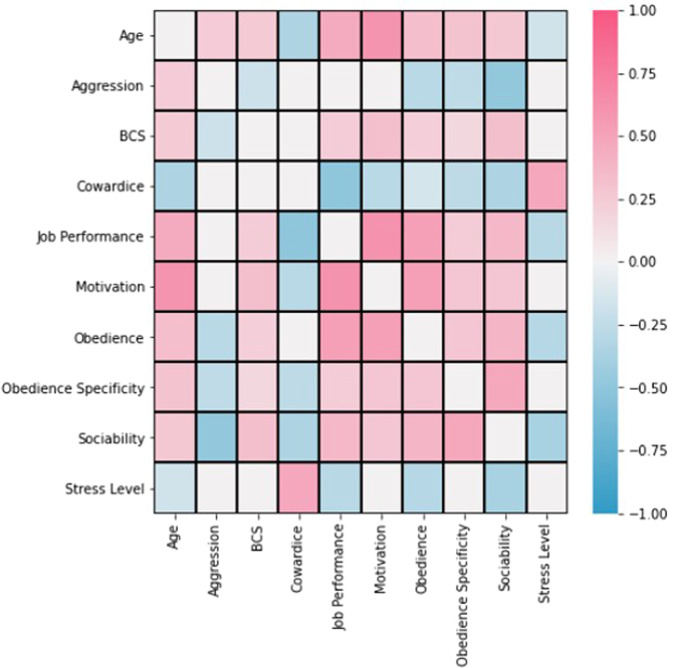
Heatmap depicting correlation among canine demographic and phenotypic data via Pearson's correlation. Only values after false discovery rate (FDR) correction (level = 0.15) are presented in this figure. *BCS Body Condition Score.

Overall scores on phenotypic outcomes including obedience, obedience specificity (the dog’s ability to be obedient to only one vs several trainers), motivation/drive, aggression, cowardice/hesitation, physical size, sociability, stress level, and job performance are presented in Table 1 and Fig. S5. Correlations among different demographic and phenotypic data were observed, for example, behaviors like obedience were positively correlated with job performance and motivation, whereas aggression was negatively correlated with sociability. Increased age was associated with increased cowardice score and increased motivation score (Fig. 6). Statistically, obedience (*p* < 0.0001), motivation (*p* < 0.0001), cowardice (*p* < 0.0001), sociability (*p* < 0.0001), obedience specificity (*p* < 0.0001), and aggression (*p* < 0.0001) scores differed based on job. Regarding specific observed trends, tracking had the largest proportion of dogs in higher obedience categories while SAR had the lowest proportion of dogs with a high score for obedience (specificity) category (i.e., SAR dogs had a lower proportion of dogs exhibiting obedience to only one trainer). Bite work, scent detection, and tracking had the highest proportions of dogs in high motivation categories. Bite work dogs had the highest proportion of dogs in high aggression categories whereas tracking had the lowest proportion of dogs in high aggression categories. SAR and scent detection had the highest proportion of dogs in the low cowardice score categories (i.e., these breeds had the highest proportion of dogs exhibiting minimal or no cowardice or hesitation). Tracking had the highest proportion of dogs in high sociability categories, whereas SAR had the lowest proportion of dogs in high sociability categories. Cowardice (*p* = 0.003), obedience specificity (*p* = 0.047), aggression (*p* = 0.003) and stress level (*p* = 0.01) scores differed based on breed. Regarding specific observed trends, Malinois and non-Malinois Dutch shepherds had higher proportions of dogs in high motivation categories. Malinois, non-Malinois Dutch shepherds, and corgis had higher proportions of dogs in high obedience categories. German shepherds, followed by Malinois and non-Malinois Dutch shepherds had the highest proportion of dogs in high aggression categories. Labradors and non-Malinois Dutch shepherds had the highest proportion of dogs in high sociability categories. A heatmap demonstrating the interactions among microbiome markers, phenotypic outcomes, and cohort appears in Fig. S4, and tables demonstrating associations between breed and behavioral outcome and job and behavioral outcome appears in Supplemental Table 2.

### Microbiome features vs quantitative phenotypes

**Fig. 7:**
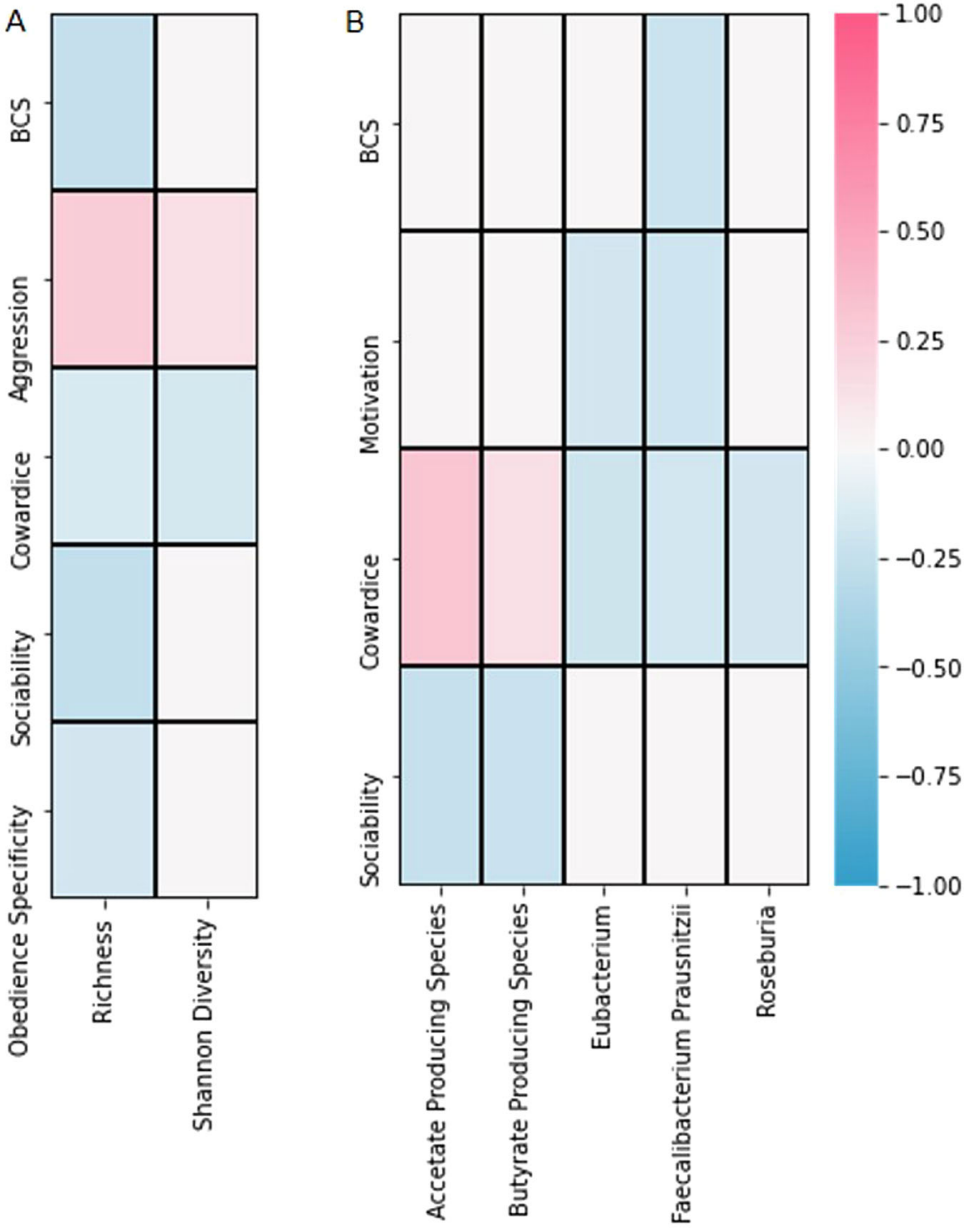
Heatmap depicting correlation among microbiome features and canine demographic and phenotypic data. **A** Microbiome features including richness and diversity. **B** Specific microbial group. Only values significant after false discovery rate (FDR) correction (level = 0.15) are presented in this figure. BCS body condition score.

**Fig. 8:**
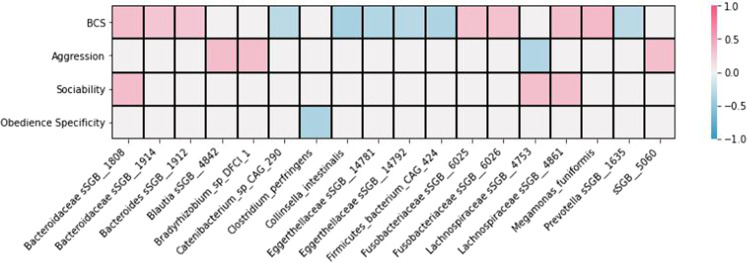
Heatmap depicting correlation among bacterial families and canine demographic and phenotypic data. Only values significant after false discovery rate (FDR) correction (level = 0.05) are presented in this figure. *BCS Body Condition Score.

When observing the correlation among microbiome features and canine demographic and phenotype data, some relationships were observed. Increased BCS was negatively associated with *Faecalibacterium prausnitzii* abundance and richness (Fig. 7), positively associated *with Bacteroides, Fusobacteriaceae, Lachnospiraceae*, and *Megamonas* abundance (Figs. 8, S6), and negatively associated with *Catenibacterium, Collinsella, Eggerthellaceae*, and *Firmicutes* abundance (Figs. 8, S6). Both positive and negative associations were observed between increased BCS and abundance of *Prevotella* (Figs. 8, S6). Increased BCS was also negatively associated with *Eubacterium* abundance (Fig. S6) and positively associated with *Selenomonadaceae* abundance (Fig. S7).

Increased motivation score was negatively associated with *Faecalibacterium prausnitzii* and *Eubacterium* abundance (Fig. 7). Increased motivation score was positively associated with *Prevotella* abundance (Figs. S6 and S7) and negatively associated with *Firmicutes* abundance (Fig. S6).

Increased cowardice score was negatively associated with *Faecalibacterium prausnitzii* abundance, *Roseburia* abundance, *Eubacterium* abundance, richness, and Shannon diversity (Fig. 7). Increased cowardice score was positively associated with butyrate-producing species (Fig. 7), acetate-producing species (Fig. 7), and *Lactobacillaceae* abundance (Fig. S7). Increased sociability scores were negatively associated with richness, abundance of butyrate-producing species, and abundance of acetate-producing species (Fig. 7). Increased sociability score was positively associated with *Lachnospiraceae* and *Bacteriodes* abundance (Figs. 8, S6) and negatively associated with *Dorea* and *Eggerthellaceae* abudance (Fig. S6). Increased aggression scores were positively associated with increased richness and Shannon diversity (Fig. 7), *Balutia* abundance (Fig. 8), *Bradyrhizobium* abundance (Figs. 8, S6), and *Ruminococcaceae* abundance (Fig. S7). Increased aggression score was negatively associated with *Lachnospiraceae* (Figs. 8, S6) and *Selenomonadaceae* abundance (Fig. S7). Increased obedience specificity scores were negatively associated with richness (Fig. 7) and *Clostridium perfringens* abundance (Fig. 8).

### Prediction of behavioral features from the microbiome

**Fig. 9:**
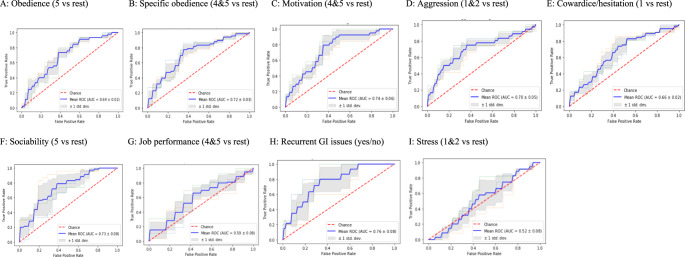
Association of phenotypic working canine characteristics with microbiome features. Classification of phenotypic characteristics was based on working canine microbiome characteristics including obedience (**A**), obedience to one trainer vs many trainers (specificity, **B**), motivation (**C**), aggression (**D**), cowardice/hesitation (**E**), sociability (**F**), job performance (**G**), recurrent gastrointestinal (GI) issues (**H**), and stress level (**I**).

For each dog characteristics such as obedience, aggression, etc. we divided our cohort into two groups with more and less exhibited characteristics. Grouping was accomplished by both observing the distribution of scores and discussing the performance and real-world implications of scores with dog trainers from the organization (Individual grouping modalities are described in Fig. 9). Then we explored the prediction from microbiome features and species abundances in order to determine to which group the sample belongs (Fig. 9). The results are shown as a mean and std of 5-fold cross-correlation accuracy. Overall, 13 microbiome features and 424 species abundances were used. Motivation, sociability, and GI issues showed the best separation and therefore suggest moderate predictive capability, and motivation and sociability had moderate separation and therefore mild to moderate predictive capability. Job performance and stress levels had the least accurate models and therefore did not show predictive capability. This suggests that there are microbiome signatures associated with certain traits in the observed dogs.

## Discussion

The overall observed abundancies were as expected, with *Firmicutes* and *Bacteroides* dominating^24,34^. Among the population as a whole, differences were observed in the microbiome by factors such as diet, adult/puppy status, sterilization status, breed, job, chronic GI problems, and BCS. This agrees with the literature, as studies have noted differences based on sterilization status^25^, breed-based differences^34,35^, working dog job^29^, GI disease^21,22,34^, diet^24,34^, and age^36^. Specific associations observed in this study also agree with the findings in the literature. For example, Scarsella et al. (2020) noted that the microbiome of sterilized dogs was less diverse than non-sterilized dogs, which agrees with our findings. However, it bears noting that most dogs with recurrent GI issues were also being fed the hypoallergenic diet so it is possible that these microbiome features are due to the influences of diet as well as GI disease (Fig. S3).

Microbiome markers were also statistically associated with motivation, aggression, cowardice, sociability, and obedience to one trainer vs many, and some of these associations agree with findings in previous studies^3,4,28^. When prediction models were developed with machine learning, the best models were for motivation, sociability, and GI issues, whereas the models that did not show predictive capability were for job performance and stress levels.

Regarding correlations between microbiome markers and behavior, some agreement with the literature was observed. We observed that increased abundance of *Firmicutes* was associated with increased aggression, which is in line with findings from Kichoff et al. (2019). Mondo et al. (2019) also observed that increased richness was associated with increased aggression and increased *Lactobacillus* was associated with increase cowardice/phobic behavior, which both agree with our findings. Interestingly our study identified increased *Ruminococcus* abundance was associated with increased aggression but not cowardice, whereas Mondo et al. (2019) observed it was associated with fearful or phobic behavior. As with Isaiah et al. (2017) higher or lower taxa abundances were associated with different canine jobs, however the taxa observed to be significant were different between our study and theirs. Nevertheless, these differences may be due to geographic location (Israel vs the United States of America) and Isaiah et al. (2017) focusing on specific subcategories within scent detection dogs vs our study which had more general groupings of a wider diversity of jobs. Finally, the ability of machine learning algorithms to predict some of these traits in samples that were held-out during the training attests to the robustness of our results.

While further research is needed to establish relationships between the canine microbiome and health, behavior, and job performance, the findings of this and previous studies strongly suggest that there is a relationship between these factors. Current studies have investigated the relationships between probiotic supplementation and diet and the canine microbiome, and outcomes of these studies combined with the findings of our study are intriguing regarding future directions for canine supplementation and dietary interventions. For example, a recent study noted that supplementing dog’s feed with probiotics resulted in positive health outcomes (including reduction of chronic diarrhea) as well as measurable shifts in numerous gut microbiome features (i.e., increase in richness, reduction in *Escherichia* spp.)^37^. Some of these features (i.e., richness, *Blautia* abundance, *Lactobacillus* abundance) were associated with health and behavioral outcomes in our study, and the fact that the microbiome shifts observed by Xu et al. (2019) were especially pronounced in elderly dogs may indicate a way to extend a dog’s healthy working years. Two other recent studies comparing dogs fed different kinds of diets noted that diet also influenced microbiome features found in our study to be associated with health and behavioral outcomes (i.e., *Prevotella, Faecalibacterium, Eubacterium*, and *Blautia* abundance)^25,38^. These concordant findings provide ample material for further longitudinal research on the effect of diet and supplementation on canine health, behavior, and performance.

Key limitations of this study are the fact that this would be considered a fairly small database for machine learning and the dogs are a non-homogenous population (i.e., diverse selection of jobs, ages, and breeds), ergo certain associations may have been masked or amplified based on these factors. Furthermore, the diversity of jobs directly impacts the desirability of specific traits. For example, an aggressive patrol or guard dog is desirable, whereas an aggressive SAR dog is not. Certain breeds, for example, Belgian Malinois, are frequently categorized as having high stress levels, and this is desirable because it translates to high drive and high performance, however high stress in a Labrador Retriever would be classified as undesirable. However, despite these factors, microbiome associations were observed between certain characteristics regardless of their desirability in that particular dog. Furthermore, a significant batch effect was observed between the two cohorts. This beta diversity difference between cohorts is potentially due to differences in breed composition by year and job by year. We have investigated the data using numerous methodologies, however, a much larger dataset would be needed to conduct multivariate statistical analysis to completely overcome this batch effect and confirm the effect of breed and job over cohort. Additionally, dogs with more than one month elapsing after antimicrobial administration were sampled, thus it is possible some lingering effects of antimicrobial administration were present in microbiome composition.

Another notable limitation is the use of an internally-relevant scale instead of an externally-validated scale. Externally-validated scales are available and could enhance future research^39,40^. However, given the variation observed in externally-validated scales^41^, the use of an internally-used scale that is well-known to the people collecting the data versus a novel-but-externally-validated scale could potentially reduce variation between cohorts^42^.

While the findings of this study point towards associations with certain microbiome features and working dog characteristics, more research is needed on this topic. For example, longitudinal research could pinpoint if microbiome shifts come before or after behavioral traits such as aggression, obedience, etc. More focused studies on one job type and/or otherwise more homogenous groups of working canines would also elucidate relationships and allow for multivariate analyses to further determine the relationships among microbiome markers and various demographic and phenotypic outcomes. As companion and working dogs are behaviorally different in terms of what is considered desirable behavior, further research should also target companion dogs. Longitudinal and job-focused studies could also allow for supplementation of pre- and pro-biotics in an attempt to ascertain if shifts in the microbiome would result in performance shifts. Importantly, our results point to specific bacterial species that associate with certain traits and thus raise concrete testable hypotheses for interventional experiments that may establish causal relationships and avenues for improving canine performance. Furthermore, for machine learning algorithms this is considered a small dataset, ergo larger studies are needed in the future to rule out batch effects.

Working canines in military and law enforcement are critical for the prevention of domestic and international criminal or terrorist acts. Their training is laborious, costly, and sometimes unsuccessful despite best efforts and all known research regarding behavior and diet. Current research has barely scratched the surface of how microbiome composition could impact performance and behavior in canines. Research findings from this study suggest potential microbiome markers for performance, and such could potentially greatly advance care for working canines. Future research should focus on the identified taxa to establish their potential as either markers of phenotypic outcomes or potentially physiological aspects that could be modulated to improve performance.

## Methods

Working canines (Total *n* = 134) from an Israeli working dog program were studied in two cohorts. Cohort 1 (*n* = 71) data were collected May–June 2018, and Cohort 2 (*n* = 63) data were collected September–December 2019. Freshly passed stools from each canine were collected using Eswabs (Copan Diagnostics, Brescia, Italy). Samples were collected from the concrete floor of regularly-cleaned individual kennels, thus reducing the risk of soil contamination. Samples were not collected from dogs that had received antibiotics in the preceding four weeks. Samples were frozen within a short timeframe (minutes to hours) until transport to the laboratory, and kept at −80° C until processing^43^. Genomic DNA was extracted using the PowerMicrobial/PowerSoil DNA kits (Qiagen, Hilden, Germany). Metadata were collected including basic demographic information (age, sex, body condition score (BCS), sterilization status, operational job, living conditions, and breed) and factors that are known to influence microbiota composition in canines (antibiotic and proton pump inhibitor (PPI) administration history, diet, history of gastrointestinal disorders). Antibiotic and PPI administration data were not available for cohort 2. BCS was assessed according to the American Animal Hospital Association scale^44^. Behavior and performance metadata (8 parameters on a scale of 1–5) were collected following assessment by dog trainers and in house veterinarians with expertize in canine behavior and work performance. These behavior and performance variables included Motivation (Dog’s drive to learn and complete tasks. Scale of 1 (no motivation, risk of failure) to 5 (highly motivated)), Aggression (Dog’s tendency to respond to stimulus (cue/command or without cue/command) with biting. Scale of 1 (no aggression, does not bite) to 5 (highly aggressive, likely to bite even when not cued/commanded)), Cowardice/hesitation (Dog’s hesitation or fearfulness regarding new or stimulating situations. Scale of 1 (no or almost no hesitation or fearfulness) to 5 (very hesitant, risk of failure)), Sociability (Dog’s social habits with other dogs. 1 = incapable of socializing with other dogs, 2 = some sociability, 3 = average sociability, can be alone or in groups, 4 = greater than average sociability, 5 = high sociability, needs group living scenario), Obedience, General (Dog’s general capability to be obedient to commands and training cues in multiple scenarios and situations. Scale of 1 (not obedient, risk of failure) to 5 (Excellent example of obedience)), Obedience specificity to one trainer vs many (Dog is obedient to any trainer using appropriate commands or cues (Score of 5), several trainers but not any trainer using appropriate commands and cues (Score of 3), or only one trainer using appropriate commands and cues (Score of 1)), Stress levels (How stressed/high strung dogs are in both day-to-day scenarios as well as job performance scenarios. Very low stress levels = 1, Not very low stress or not stressed only in some scenarios = 2, average stress levels = 3, Not very high stress/highly stressed in some scenarios = 4, Very stressed in all scenarios = 5), and Job performance (Scale of 1 (bad at assigned job, risk of failure) to 5 (Excellent example of canine performance in assigned job)).

Whole genome sequencing was performed using the Illumina NextSeq 500 platform (Illumina, San Diego, CA). For metagenome analysis, metagenomic reads containing Illumina adapters and low-quality reads were filtered with trimming of low-quality read edges. Host DNA was detected by mapping with bowtie to the *Canis lupus familiaris* genome^45^ with inclusive parameters, and host reads were removed. We subsampled to 10 million reads. Relative abundances from metagenomic sequencing were computed using our developed relative abundance estimation as described below. Relative abundances were capped at a level of 10^−4^.

The bacterial reference dataset for relative abundance estimation was based on the representative assembly of the species-level genome bins (SGBs) and genus-level genome bins (GGBs) defined by Pasolli et al.^46^. Human SGBs were utilized for this stage of analysis; out of the 4930 human SGBs (associated with various body sites), 3127 SGBs were used, which were characterized by either belonging to a unique genus or with at least 5 assemblies to justify having a new SGB. We employed this restriction, since we noticed that the cutoff threshold used by Pasolli et al. to cluster assemblies into SGBs, resulted in the artificial splitting of small groups with little nucleotide difference from a large nearby SGB. Abundance was calculated by counting reads that best match to a single SGB. Bowtie2^47^ was used to map samples from our cohort versus an index built from representatives of the SGBs. When analyzing the mapping, we focused on reads for which the best map is unique (thus mapped to a location which is unique in the index of representatives). We counted the number of reads uniquely mapped to each window of each SGB. The cover estimation for each SGB is the dense mean cover of its representative^48^, normalized by the genome size. The relative abundance estimation is the cover divided by the sum of the covers of all representatives concluded to exist in this sample. The microbiome features of butyrate-producing species were calculated by taking the sum of the abundances of butyrate-producing bacteria based on the literature (24 bacteria species total)^49^. The microbiome feature of *Firmicute/Bacteroides* ratio was calculated by dividing the sum of bacteria abundances from the phylum *Firmicutes* by the sum of bacteria abundances from the phylum *Bacteroides*.

For descriptive statistics and overall evaluation of beta diversity features, R (Version 4.0.2) was used with the following packages: tidyverse, dplyr, phyloseq, data.table, devtools, microbiomeutilities, readr, ggplot2, and psych. Chi squared and fisher’s exact tests were used to evaluate demographic trends as well as correlations between phenotypic/behavioral outcomes and demographic inputs.

To evaluate the discriminative power of the microbiome composition, we constructed a prediction model of behavioral/phenotypic outcomes using gradient boosting classification which takes the microbiome features as inputs for (feature) based on Xgboost^50^. Young dogs that would not have yet reached emotional and behavioral maturity (<1 year of age) were not included in this analysis. The mean and standard deviation of the area under the receiver operating characteristic (ROC AUC) curve were computed by using the curves that were generated in 3-fold cross-validation. The following python (v 3.7.2) libraries were used for statistics (scipy ‘1.5.4’), visualization (seaborn 0.11.0), and prediction (xgboost 1.3.0). Specifically, regarding comparisons of microbiome features and phenotypic/behavioral outcomes, Kruskal–Wallis and Mann–Whitney tests were used. Pearson correlation was used to compare between two numerical features and Bonferroni correction was applied when testing multiple hypotheses (on the level of 0.15).

### Reporting summary

Further information on research design is available in the Nature Research Reporting Summary linked to this article.

## Supplementary information

Reporting Summary

Supplementary Materials

## Data Availability

The datasets generated during the current study are available in BioProject repository, under project number PRJEB45252.

## Code availability

Code is available from the authors upon request.
