# Supplementary material for: Phenotypic correlates of the working dog microbiome
Source: NPJ Biofilms Microbiomes. 2022 Aug 22;8:66. doi: 10.1038/s41522-022-00329-5 (PMC9395329; doi:10.1038/s41522-022-00329-5)

SUPPLEMENTARY MATERIALS

Title: Phenotypic Correlates of the Working Dog Microbiome

Authors: Hillary A. Craddock *[1], Anastasia Godneva *[2], Daphna Rothschild [2], Yair Motro [1], Dan Grinstein [1], Yuval Lotem-Michaeli [1], Tamar Narkiss [3], Eran Segal [2] and Jacob Moran-Gilad [1]**

Author Affiliations

1. MAGICAL Group, Department of Health Systems Management, School of Public Health, Faculty of Health Sciences, Ben-Gurion University of the Negev, Beer-Sheva, Israel.
2. Department of Computer Science and Applied Math, Weizmann Institute of Science, Rehovot, Israel
3. Israel Defense Forces Medical Corps, Tel Hashomer, Ramat Gan, Israel

* These authors contributed equally to the work

**Corresponding author, E-mail: giladko@post.bgu.ac.il

Supplementary Table 1: Mean abundance of major taxa in Cohort 1 vs Cohort 2 of working dogs.

	Cohort 1		Cohort 2	
**Taxa**	**Mean abundance**	**StDev**	**Mean abundance**	**StDev**
Actinobacteria	0.0129	0.0115	0.0949	0.0674
Bacteroidetes	0.563	0.181	0.269	0.18
Candidatus_Melainabacteria	0.000158	0.000245	0.000101	0.00000454
Firmicutes	0.32	0.18	0.577	0.18
Fusobacteria	0.0463	0.0346	0.0245	0.0282
Proteobacteria	0.058	0.0966	0.0398	0.0553
unknown	0.0355	0.0409	0.0267	0.0244

Supplementary Table 2: Proportion of dogs in low or high scoring categories, by job (Panel A) and by breed (Panel B). Relationships that were found to be statistically significant are presented here.

A)

**Job**	Obedience		Motivation		Cowardice		Sociability		Obedience (specificity)		Agression	
	Low score	High score	Low score	High score	Low score	High score	Low score	High score	Low score	High score	Low score	High score
Bite work	0.13	0.87	0.13	0.87	0.80	0.20	0.60	0.40	0.20	0.80	0.20	0.80
Breeding	0.52	0.48	0.52	0.48	0.65	0.35	0.43	0.57	0.00	1.00	0.74	0.26
Failed	0.86	0.14	0.86	0.14	0.00	1.00	0.71	0.29	0.14	0.86	0.71	0.29
SAR	0.53	0.47	0.53	0.47	0.33	0.67	0.73	0.27	0.67	0.33	0.67	0.33
Scent detection	0.28	0.72	0.28	0.72	0.22	0.78	0.44	0.56	0.17	0.83	0.78	0.22
Tracking	0.11	0.89	0.11	0.89	0.69	0.31	0.11	0.89	0.14	0.86	0.86	0.14

B)

**Breed**	Cowardice		Obedience (specificity)		Agression		Stress Level	
	Low score	High score	Low score	High score	Low score	High score	Low score	High score
Belgian Dutch	0.36	0.64	0.21	0.79	0.79	0.21	0.57	0.43
Corgi	0.75	0.25	0.17	0.83	0.92	0.08	0.75	0.25
GSD	0.44	0.56	0.38	0.63	0.25	0.75	0.69	0.31
Labrador	0.75	0.25	0.38	0.63	1.00	0.00	0.88	0.13
Malinois	0.52	0.48	0.19	0.81	0.63	0.37	0.69	0.31
Other	0.45	0.55	0.18	0.82	1.00	0.00	1.00	0.00

* GSD = German Shepherd Dog

** SAR = Search and Rescue

Supplementary Figure 1: Relative abundance of major taxa in working dogs, grouped by cohort.

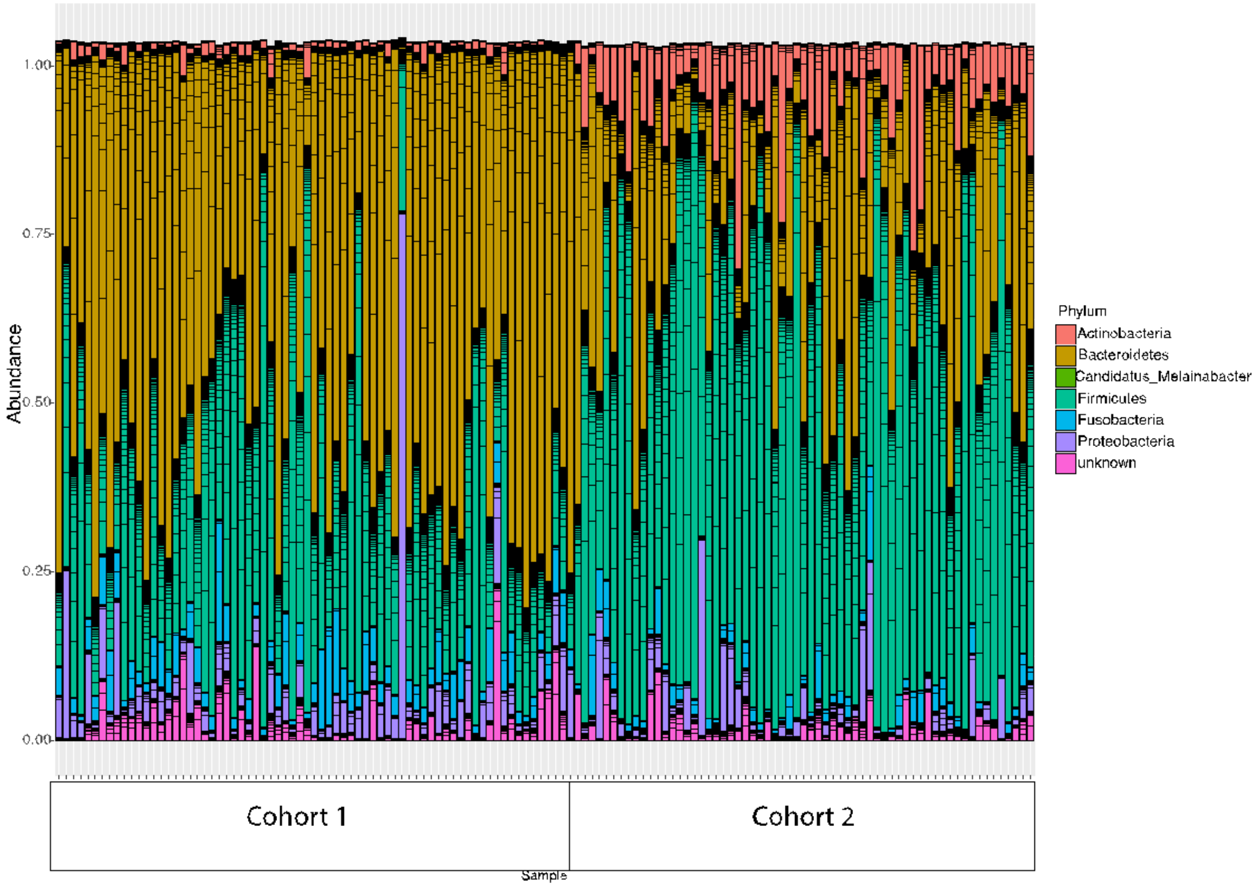

Supplementary Figure 2: PCoA plots grouped by breed (shape) and cohort (Cohort 1 = black, Cohort 2 = blue) in Panel a and job (shape) and cohort (Cohort 1 = black, Cohort 2 = blue) in Panel b.

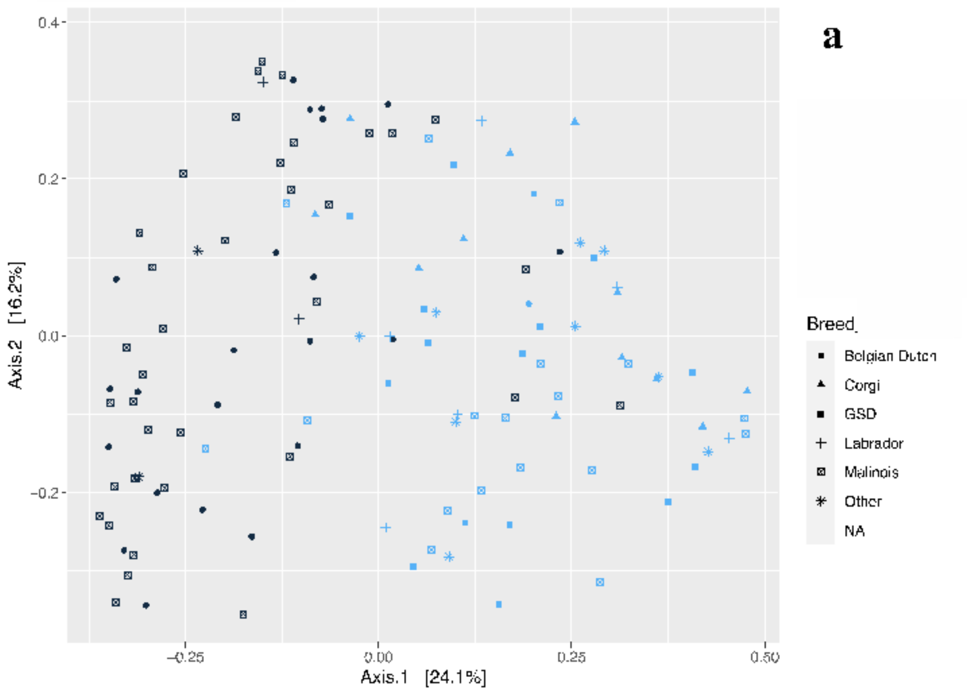

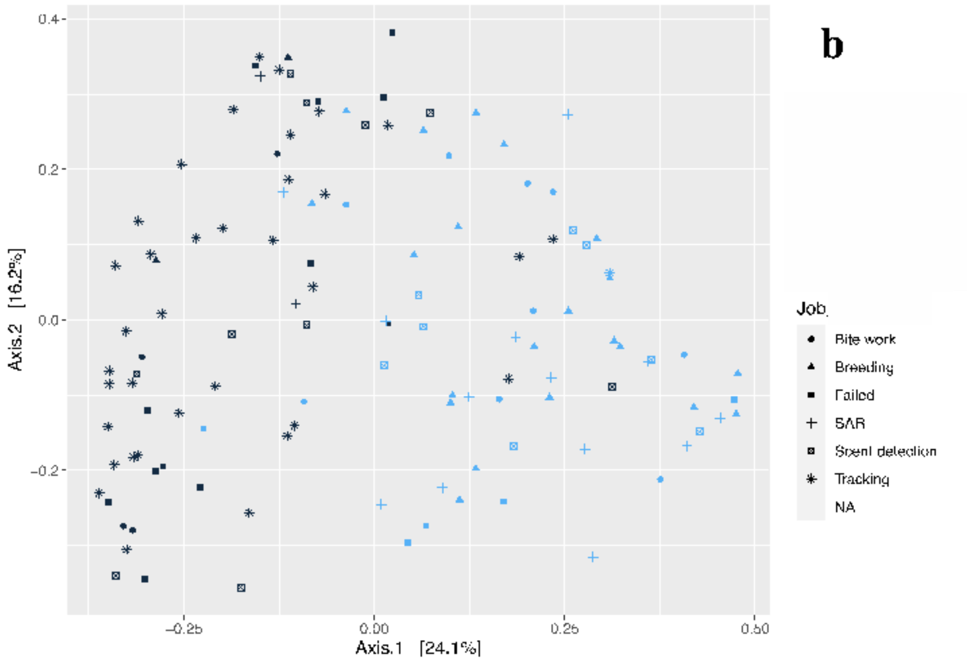

Supplementary Figure 3: Relative value of microbiome features that differ by canine diet and are statistically significant (by kruskal test, $p<0.05$). Panel A reflects differential value of richness and diversity, and Panel B reflects relative abundance. Error bars represent confidence intervals.

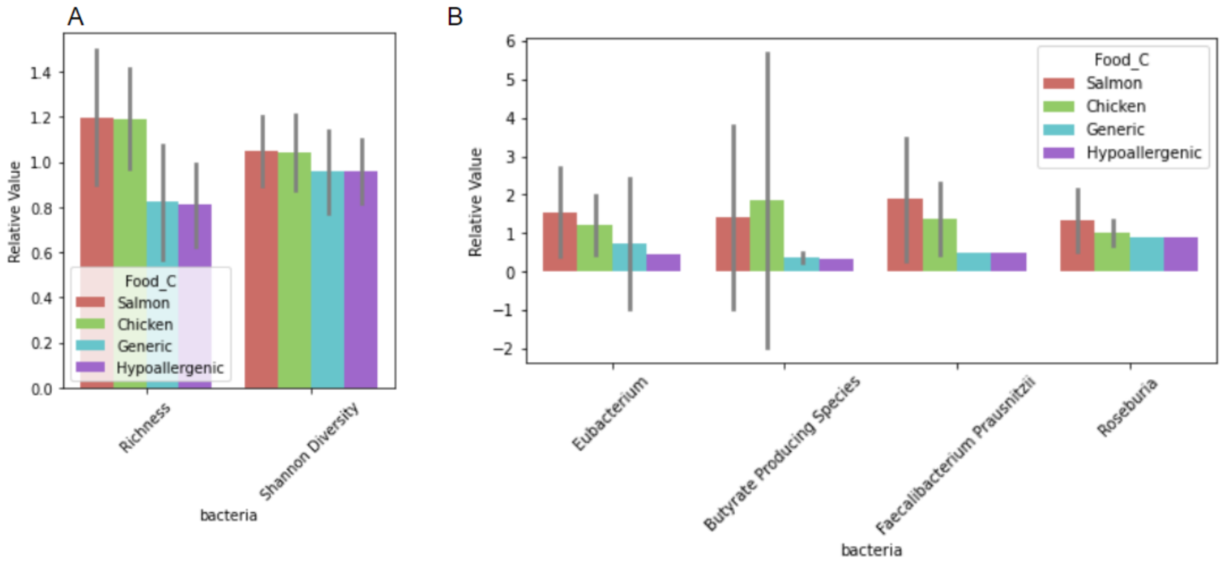

Supplementary Figure 4: Heatmap demonstrating microbiome markers differentiated based on year (Panel A) and concurrence with other demographic and behavioral characteristics and year (Panel B).

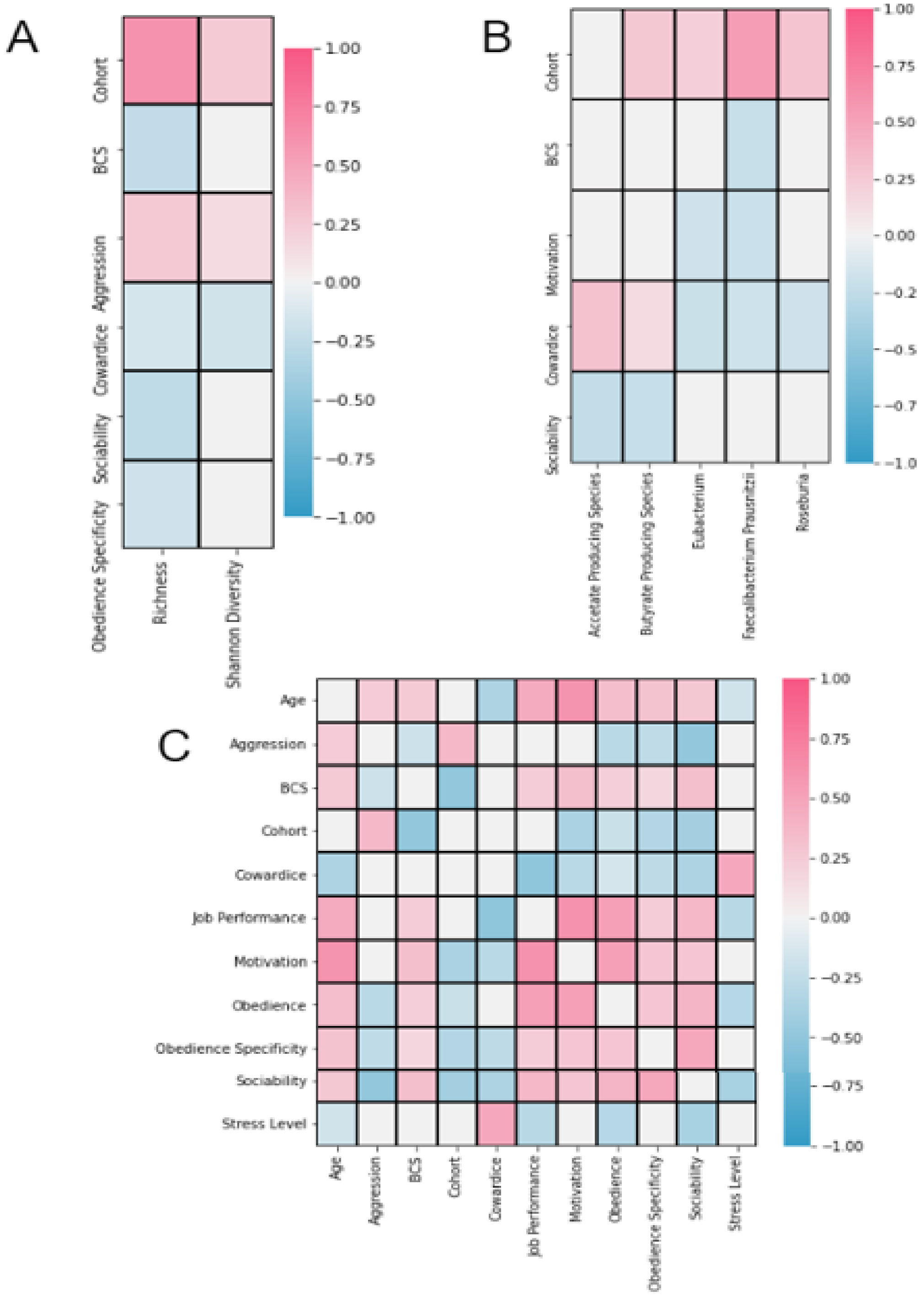

Supplementary Figure 5: Histograms of observed phenotypic/behavioral outcomes for both cohorts of working dogs combined.

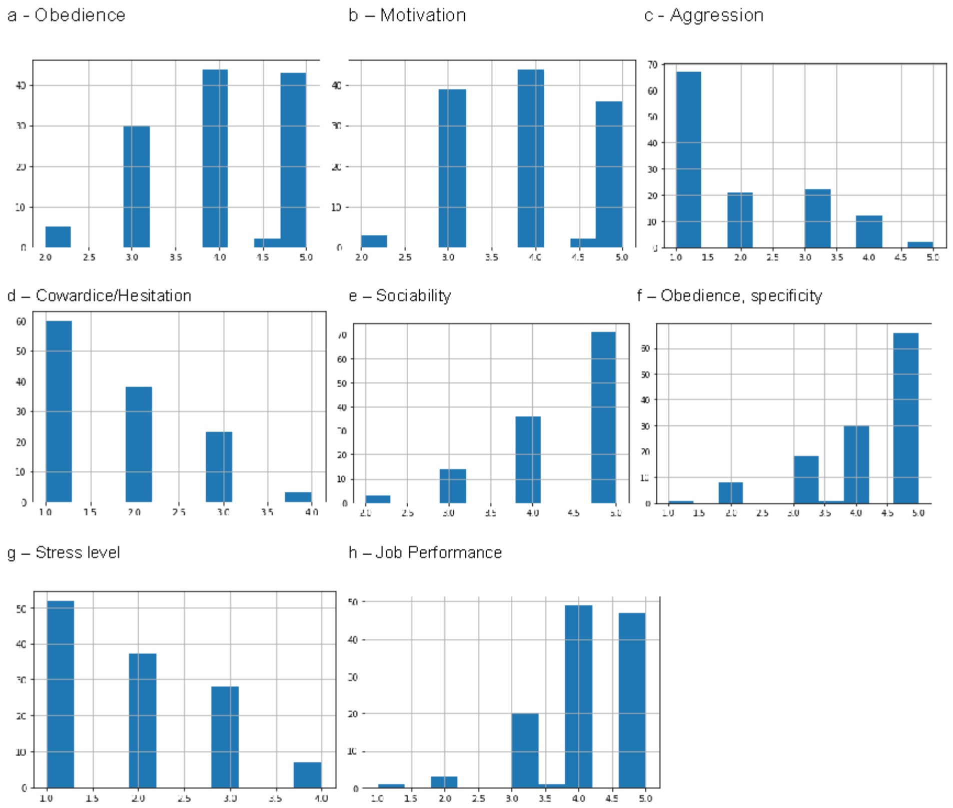

Supplementary Figure 6: Heatmap depicting correlation among bacterial genus and canine demographic and phenotypic data. Only values significant after false discovery rate (FDR) correction (level = 0.05) are presented in this figure.

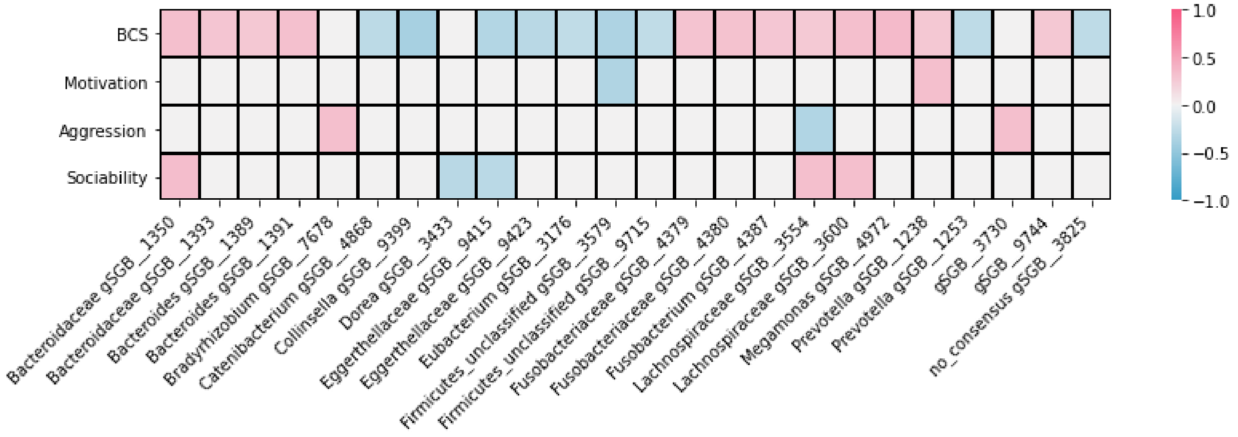

Supplementary Figure 7: Heatmap depicting correlation among bacterial species and canine demographic and phenotypic data. Only values significant after false discovery rate (FDR) correction (level = 0.05) are presented in this figure.

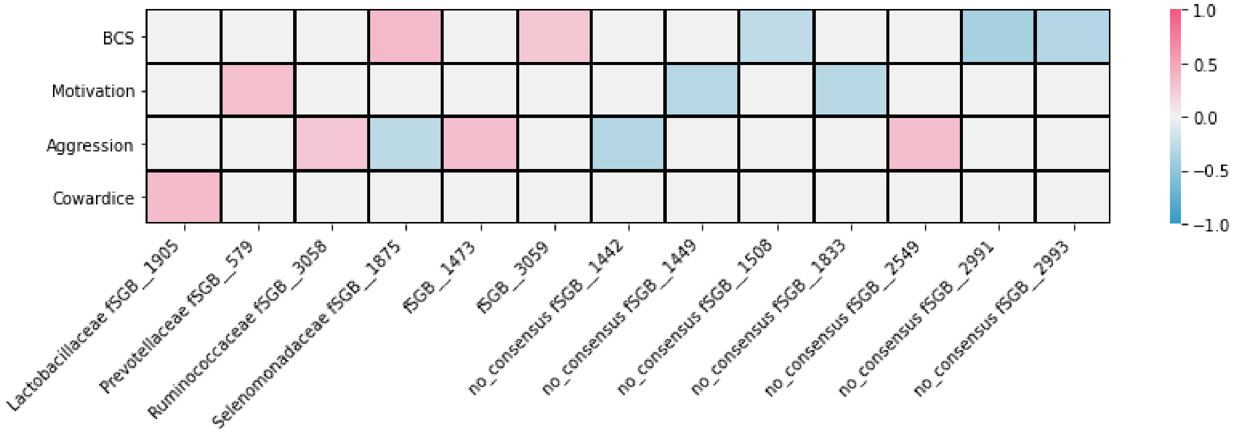

Supplement: Supplementary file 2 — Supplementary Materials [file 41522_2022_329_MOESM2_ESM.pdf]
